# Comparison of serum metabolomics in women with breast Cancer Prior to Chemotherapy and at 1 year: cardiometabolic implications

**DOI:** 10.1186/s12905-023-02355-7

**Published:** 2023-05-03

**Authors:** Debra E. Lyon, Yingwei Yao, Timothy Garrett, Debra Lynch Kelly, Lakeshia Cousin, Kellie J. Archer

**Affiliations:** 1 College of Nursing, University of Florida, Gainesville, USA; 2 College of Medicine, University of Florida, Gainesville, USA; 3 College of Public Health, the Ohio State University, Columbus, USA

**Keywords:** breast cancer, survivorship, metabolomics, inflammation

## Abstract

**Objective:**

Early-stage breast cancer (BC) is the second most common malignancy in women, worldwide. Early-detection and treatment advances have led to 5-year survival rates of 90% for early-stage breast cancer. However, the long-term morbidity of breast cancer remains high, with a majority of survivors facing increased risk of cardiometabolic conditions as well as secondary cancers. In particular, African American women with breast cancer experience higher morbidity and mortality than other women. Metabolomics is the comprehensive study of metabolites in biological samples to elucidate the role of monosaccharides, amino acids, and their respective metabolic pathways. Although some studies have found differential metabolites in women with breast cancer compared to normal controls, there has been little study of women with breast cancer across time and the active treatment trajectory. This study examines and compares the serum metabolomic profile of women with BC, prior to initial chemotherapy and at 1 year after inception of chemotherapy.

**Methods:**

This study examined serum metabolites through a secondary analysis of a longitudinal parent study (EPIGEN) of women diagnosed with early-stage BC. Participants were evaluated across 5 time points: prior to their receipt of chemotherapy (T1), at the time of their 4th chemotherapy treatment (T2), 6 months after the initiation of chemotherapy (T3), one year after the initiation of chemotherapy (T4) and two years after the initiation of chemotherapy (T5). This analysis focused on the metabolomic data from 70 participants from T1 to T4. Using ultra high-pressure liquid chromatography high resolution mass spectrometry (UHPLC-HRMS), we performed Friedman Rank Sum Test followed by Nemenyi post-hoc pairwise tests to identify which metabolite levels differed between time points, focusing on metabolites with a Benjamini-Hochberg false discovery rate (FDR) from the overall Friedman test < 0.05 and then specifically examined the p-values from the T1 vs. T4 pairwise comparison.

**Results:**

The untargeted serum metabolomics yielded a total of 2,395 metabolites identified on the basis of the accurate mass and MS/MS fragmentation, 1,264 of which were significant after Friedman’s test (FDR < 0.05). The analysis then focused on the levels of 124 metabolites from the T1 vs. T4 post-hoc comparison that had a combined FDR < 0.05 and fold change (FC) > 2.0. Metabolite set enrichment analysis (MSEA) as part of Metaboanalyst 3.0 was performed to identify pathways that were significantly altered. The known metabolites identified from the functional analysis were used to evaluate the up and down regulated pathways. The 40metabolites from the Functional Analysis were mainly attributed to amino acids (specifically lysine regulation), fatty acids (particularly unsaturated) and steroid hormone synthesis (lysophosphatidic acid).

**Conclusion:**

There were multiple significant changes in the serum metabolomic profile of women with breast cancer at one-year post inception of chemotherapy compared to pre-chemotherapy, most notably associated with lysine degradation, branched-chain amino acid synthesis, linoleic acid metabolism, tyrosine metabolism and biosynthesis of unsaturated fatty acids as the top 5 metabolic pathways. Some of these changes could be associated with metabolic perturbations that are consistent with heightened risk of cardiometabolic morbidity. Our results provide new insights into the mechanisms underlying potential heightened cardiovascular health risks in this population.

## Introduction

Early-stage breast cancer (BC) is the second most common malignancy in women, worldwide. Early detection and treatment advances have led to five-year survival rates of 90% across all stages of breast cancer [1]. However, an increasing challenge in breast cancer treatment is a better understanding of the heightened risk for non-cancer related excessive morbidity and mortality post-treatment, particularly for Black women, who experience a 41% higher mortality risk than White women [2]. Not only are there adverse racial disparities in breast cancer outcomes, there is increased cardiovascular (CVD) mortality among Black women, particularly those diagnosed with cancer at younger ages [3], yielding overlapping disparities in CVD and breast cancer observed by race and ethnicity [4].

Innovations in metabolomics permit understanding of how metabolism may change over time in the first year of breast cancer treatment. This knowledge could provide a basis for better understanding elevated survivorship risks and lead to personalized strategies for mitigating health risks. Metabolomics is the comprehensive global study of metabolites such as monosaccharides, amino acids, acylcarnitines, organic acids, purines and many others in biological samples to elucidate their roles in disease individually and through their respective metabolic pathways [5]. Metabolomics reflects molecular processes more proximal to disease states than other ‘omic’ markers. Nontargeted metabolomics is a comprehensive analytic approach that attempts to detect, identify, and relatively quantify as many metabolites in a biological sample as possible, thus presenting a discovery technique for the identification of individual or patterns of metabolic alterations associated with a particular phenotype [6]. Although some studies have found differential metabolites in women with breast cancer compared to normal controls, there has been little study across time and the treatment trajectory of women treated for breast cancer. To better understand metabolite changes across the treatment trajectory and into survivorship, this study examines and compares the serum metabolomic profile of women with BC, prior to initial chemotherapy and at 1 year after inception of chemotherapy. Elucidation of metabolic alterations could help in understanding of the pathophysiological processes associated with heightened cardiovascular risks in breast cancer survivors.

## Materials and methods

### Ethics statement

This research involving human subjects was approved by the Virginia Commonwealth University Human Subjects’ Institutional Review Board (IRB # HM13194 CR4) and University of Florida RB201400083. Written documentation of informed consent was obtained from all study participants. All mandatory laboratory health and safety procedures have been complied with in the course of conducting any experimental work reported in this paper. In the parent study, 77 women with early stage (I to IIIA) BC were ascertained through 5 regional cancer centers in Central Virginia as part of the EPIGEN study, as previously described [7, 8]. Participant eligibility criteria were as follows: (1) 21 years of age or older, (2) a diagnosis of early stage BC with a scheduled visit to receive chemotherapy, and (3) female (due to the low frequency of BC in males, only females were evaluated). Exclusion criteria were a history of (1) a previous cancer or chemotherapy, (2) a diagnosis of dementia, (3) active psychosis, or (4) an immune-related diagnosis (to avoid confounding due to inflammation). The five time-points in the parent longitudinal study were as follows: time-point 1 (“baseline” time-point, which was prior to the inception of chemotherapy), time-point 2 (“mid-chemo” time-point, which was prior to the fourth cycle of chemotherapy), time-point 3 (approximately 6 months following the inception of chemotherapy, at which time a subset of women received radiotherapy), and time-points 4 and 5, (approximately 1 year or 2 years following chemotherapy inception, respectively). Participants received either “dose dense” chemotherapy (every 2 weeks) or had an every 3-week chemotherapy administration (based on their regimen). All participants completed chemotherapy prior to time-point 3. After completion of chemotherapy, women with hormone sensitive tumors began hormonal agents. During each time-point visit, blood specimens were collected from each study participant and transported to the laboratory and stored using standard laboratory protocols. For each study participant, demographic and lifestyle information was obtained (via self-reporting at time-point 1), along with clinical health information (extracted from the electronic health record following assessments by a research nurse). In this analysis, we examined serum metabolomics at T1 and T4 timepoints to characterize differences prior to treatment compared to one year after the inception of breast cancer treatment and into survivorship.

### Procedures

Analysis of metabolites was conducted on a Thermo Q-Exactive mass spectrometer with a Dionex 3000 UHPLC in both positive and negative electrospray ionization. Separation prior to mass analysis was achieved on a Waters Atlantis HILIC (150 × 2.1 mm, 3 μm) with mobile phase A as 20 mM ammonium formate adjusted to pH 4 with acetic acid and mobile phase B as acetonitrile with 0.1% acetic acid. Initial conditions are 95% B held for 1 min, then decrease B to 50% for 9 min, holding for 4.5 min before returning to initial conditions for 0.5 min and equilibrating for 4 min. The flow rate was 0.3 mL/min with a column temperature of 30 °C. 25 µL of sample was aliquoted and 5 µL of internal standard solution was added followed by 200 µL of 8:1:1 acetonitrile:methanol:acetone. The samples were vortexed and incubated for 30 min at 4 °C. The samples were then centrifuged at 20,000 rcf (4 °C) for 10 min. Next, 200 µL of the supernatant was transferred to a new tube and dried under a gentle stream of nitrogen (30 °C). The samples were reconstituted with 25 µL of injection standard solution prepared in 90:10 acetonitrile:10 mM ammonium acetate. Solvent blanks and extraction blanks were also prepared.

### Metabolite identification

Metabolite alignment was conducted with MZmine 2.53 following an automated process developed in the lab [9]. Features were filtered using blank feature filtering to remove signals below the noise threshold [10]. Adducts were identified by exact mass and retention time correlation and removed from the data set. Metabolite identification was conducted using the Functional analysis feature of Metaboanalyst 3.0 [11, 12]. A spreadsheet of the *m/z* value, the retention time, the polarity of ionization and the fold change were used for metabolite identification in Functional Analysis with a mass tolerance of 10 ppm. Only metabolites that were significant (FDR < 0.05) were used for Functional analysis. Additional metabolites were identified by matching to an in-house library by *m/z* and retention time. These are annotated in capital letters.

### Statistical analysis

The analyses were restricted to the 70 subjects having T1 – T4 data available. Demographic and treatment characteristics were summarized by reporting the mean (SD) for continuous variables and reporting frequencies with percentages for categorical variables. Because of anticipated non-linearities we performed Friedman Rank Sum Test followed by Nemenyi post-hoc pairwise tests to determine which metabolite levels differed among and between timepoints. We restricted the metabolites to those having a Benjamini and Hochberg false discovery rate (FDR) from the overall Friedman test < 0.05 and then specifically examined the p-values from Nemenyi post-hoc pairwise tests for the T1 vs. T4 pairwise comparison.

## Results

In total, 77 women with early stage BC were recruited for the parent study. Two participants withdrew prior to initial data collection. There were 75 unique patients at Time 1, 75 at Time 2, 73 at Time 3, and 70 at Time 4. We restricted the current study to the 70 participants having metabolomics assays performed at all four timepoints. Only two women (one in the Black cohort; one in the White cohort) self-reported having Latino/Hispanic ethnicity. Given this small number, no statistical analyses were performed for the Latino/Hispanic sub-group, with these women being included in the Black or White sub-groups, respectively. The age of the 70 women in the study ranged from 23 to 71 years, with a median age of 52 years. A significant difference in age was observed between the Black (mean = 46.2 years, standard error (s.e.) = 1.94 years) and White (mean = 53.9 years, standard error (s.e.) = 1.45 years) participants (*p* = 0.003). Demographic data, breast tumor characterizations, and treatment information for these 70 women are shown in Table 1.

**Table 1:** Demographics of the Sample (N = 70)

Age (mean [SD])		51.61 (10.34)
Ethnicity (%)	Hispanic or Latino	3 (4)
	Not-Hispanic or Latino	67 (96)
Race (%)	Black or African American	21 (30)
	White	49 (70)
Education (%)	Didn’t finish High School	7 (10.0)
	High School Diploma	8 (11)
	Any education beyond High School	55 (79)
Employment (%)	Disabled	5 ( 7)
	Full-time	38 (54)
	Part-time	5 ( 7)
	Retired	10 (14)
	Student	1 ( 1)
	Unemployed	11 (16)
Income (%)	Less than 15,000	11 (16)
	Between 15,000 and 29,999	5 ( 7)
	Between 30,000 and 44,999	8 (11)
	Between 45,000 and 59,999	7 (10)
	Between 60,000 and 74,999	7 (10)
	Between 75,000 and 89,999	10 (14)
	Between 90,000 and 104,9999	8 (11)
	Greater than or equal to 105,000	14 (20)
Marital Status (%)	Divorced/Separated	14 (20)
	Married/Partner	46 (66)
	Single never married	10 (14)
Hours of Sleep (mean (SD))		6.65 (1.57)
Menstrual Status (%)	Peri-menopausal	6 (9)
	Post-menopausal	40 (57)
	Premenopausal	24 (34)
Currently Smoking (%)	No	57 (81)
	Yes	13 (19)
Current ETOH (%)	No	31 (44)
	Yes	39 (56)
Weight (mean (SD))		171.87 (39.2)
Height (mean (SD))		63.48 (3.56)
BMI (mean (SD))		30.21 (7.56)
Grade (%)	1	5 ( 7)
	2	28 (40)
	3	37 (53)
Luminal A (%)	N	33 (47)
	Y	37 (53)
Luminal B (%)	N	64 (91)
	Y	6 ( 9)
Triple negative (%)	N	50 (71)
	Y	20 (29)
Estrogen receptor positive (%)	N	29 (41)
	Y	41 (59)
TX for ER (%)	Anastrozole, 1 mg, daily x 2years	1 ( 1.5)
	Anastrozole, 1 mg, dailyx2years	5 ( 7.7)
	Arimidex 1 mg 1PO daily x 2 years	2 ( 3.1)
	Arimidex 1 mg 1PO daily x 5 years	5 ( 7.7)
	Arimidex 1 mg 1PO daily x 5years	1 ( 1.5)
	Femara 2.5 mg 1PO daily x 5 years	2 ( 3.1)
	Letrozole 2.5 mg 1PO daily x 5 years	1 ( 1.5)
	Letrozole 2.mg daily x 9 months	1 ( 1.5)
	None	30 (46.2)
	Tamoxifen 20 mg daily x 5 years	1 ( 1.5)
	Tamoxifen 20 mg 1PO daily x 5 years	3 ( 4.6)
	Tamoxifen 20 mg daily	1 ( 1.5)
	Tamoxifen 20 mg daily x 5 years	5 ( 7.7)
	Tamoxifen, 20 mg, daily x 10 years	3 ( 4.6)
	Tamoxifen, 20 mg, daily x 10 years	1 ( 1.5)
	Tamoxifen, 20 mg, daily x 5 years	3 ( 4.6)
Progesterone Receptor positive (%)	N	33 (47)
	Y	37 (53)
HER2 pos (%)	N	57 (81)
	Y	13 (19)
HER2 pos ER/PR neg (%)	N	63 (90)
	Y	7 (10)
TX for HER pos (%)	6 mg/kg every 3 weeks x1 year	1 ( 1.4)
	Herceptin 2 mg/kg IV times 12 weeks to be followed by maintenance Herceptin for 1 year	1 ( 1.4)
	Herceptin 6 mg/kg IV q3 weeks x 1 year	2 ( 2.9)
	Herceptin 6 mg/kg every 3 weeks x 1 year	4 ( 5.8)
	Herceptin 6 mg/kg every 3 wks	1 ( 1.4)
	Herceptin 6 mg/kg every three weeks x 1 year	2 ( 2.9)
	Herceptin 6 mg/kg q3 weeks	1 ( 1.4)
	Herceptin 6 mg/kg q3 weeks x 1yr	1 ( 1.4)
	None	56 (82.1)
Number of Lymph Pos (mean (SD))		0.96 (2.15)
Surgery (%)	Biopsy	5 (7)
	Lumpectomy	20 (29)
	Segmental	14 (20)
	Simple	30 (44)
Neoadjuvant (%)	N	63 (90)
	Y	7 (10)
Chemo Final (%)	AC	2 ( 2.9)
	CMF	2 ( 2.9)
	TAC	34 (48.6)
	TC	20 (28.6)
	TCH	12 (17.1)
Followed by Taxane (%)	N	37 (55.2)
	Y	30 (44.8)
Radiation TX (%)	N	16 (22.9)
	Y	54 (77.1)

**Fig. 1:**
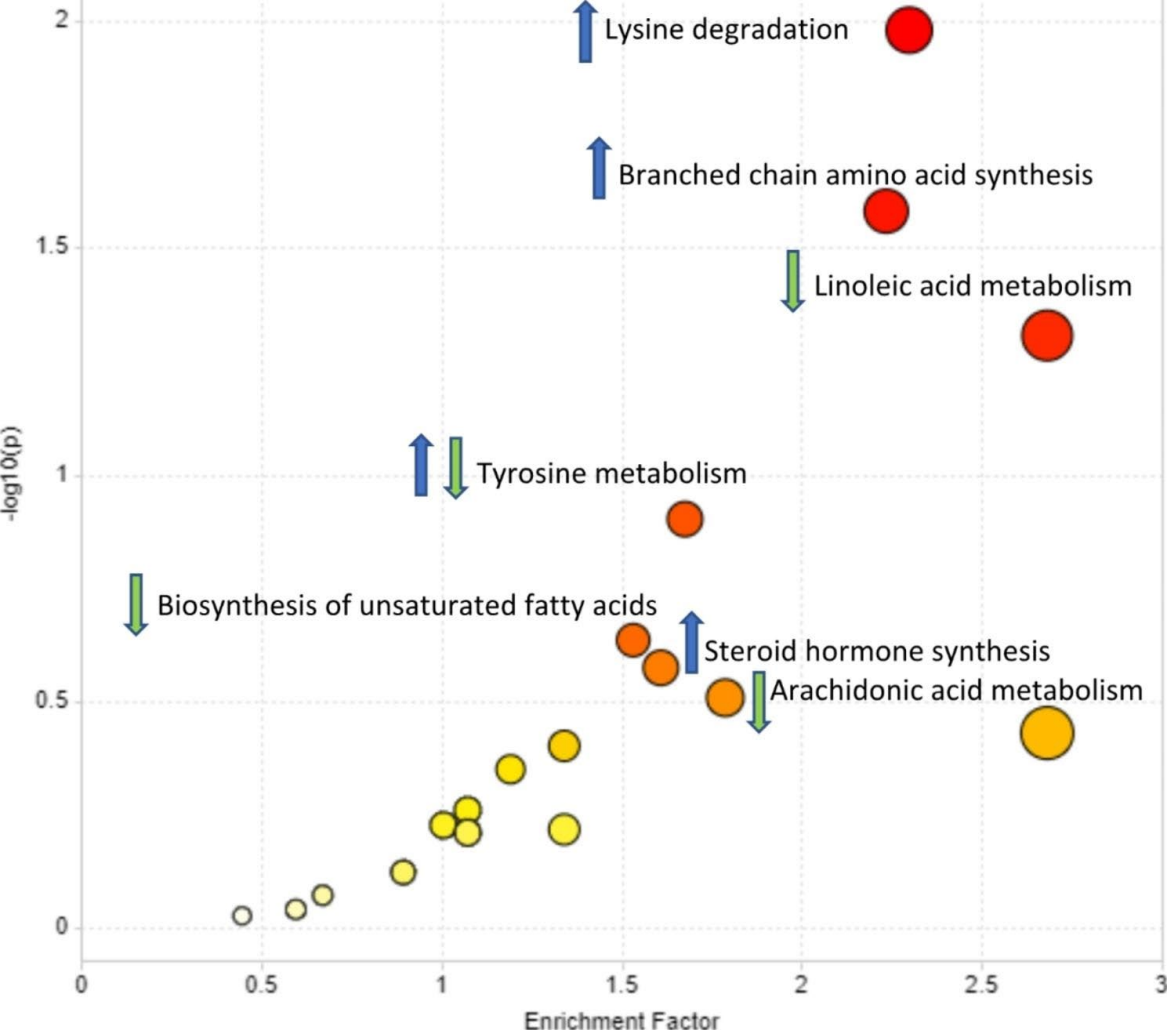
Metabolite Set Enrichment Analysis (MSEA) showing the pathways affected. Pathways that were upregulated are indicated with a blue arrow while pathways that were downregulated are indicated with a green arrow. Lysine degradation and branched chain amino acid synthesis were the most significant upregulated pathways. The tyrosine pathway was both up and down regulated

**Fig. 2:**
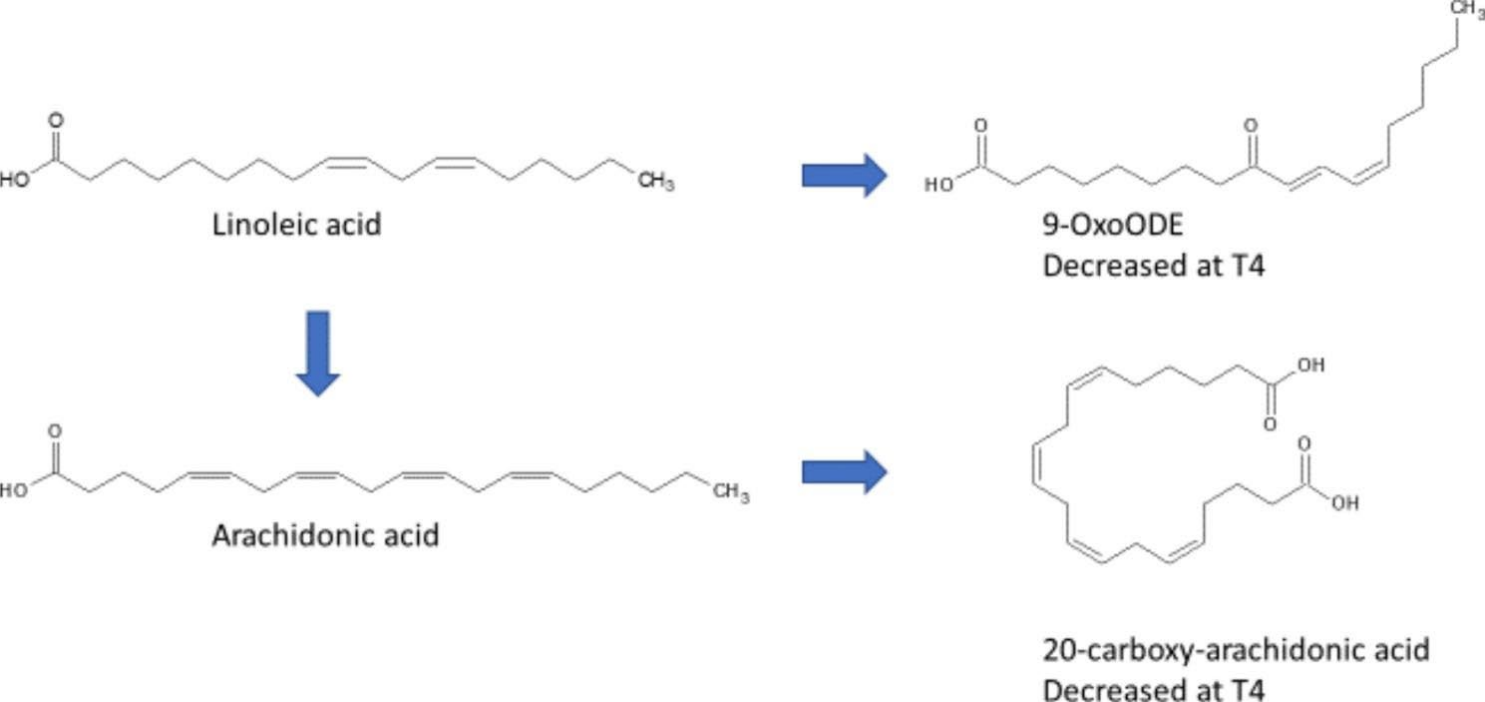
Linoleic acid metabolism signaling pathways

The untargeted metabolomics data of serum yielded a total of 2,395 metabolites identified on the basis of the accurate mass and MS/MS fragmentation. Then after performing Friedman’s test, there were 1,264 that were significant using an FDR < 0.05, of which 723 showed a differential expression between T1 and T4 (p < 0.05; FDR < 0.05). This was after blank feature filtering, adduct and complex removal and duplicate removal. The analysis then focused on the levels of 124 metabolites from the T1 vs. T4 post-hoc comparison that had a combined FDR < 0.05 and fold change (FC) > 2.0. Metabolite set enrichment analysis (MSEA) as part of Metaboanalyst 3.0 was performed to identify pathways that were significantly altered. The known metabolites identified from the Functional analysis were used to evaluate the up and down regulated pathways. We were then able to identify 40 metabolites from the Functional Analysis: 28 unique metabolites were up-regulated at T4 (Table 2) and 12 were down regulated at T4 (Table 3). The identified metabolites were mainly attributed to amino acids (specifically lysine regulation), fatty acids (particularly unsaturated) and branched chain amino acids (Fig. 1) and linoleic acid pathways (Fig. 2).

**Table 2:** Metabolites concentrations increased from T1 to T4

Metabolite name	Metabolite	Mean baseline	Mean time 4	Baseline vs. Time 4 *p* value	Time4 Baseline (FC)	Log p Value	m/z	RT
Diallylamine	4078 98.0964 7.99 NA P	11583397.44	34950234.03	3.74E-14	3.02	13.43	98.0964	7.99
N,N,N-Trimethylethenaminium	41,408 86.0966 7.7 NA P	6800588.49	19335002.09	5.62E-14	2.84	13.25	86.0966	7.7
2-Imino-4-methylpiperidine	4634 113.1071 6.92 NA P	3687620.29	8959504.60	6.22E-14	2.43	13.21	113.107	6.92
3-hydroxyheptanoylcarnitine	5479 272.1859 1.53 NA P	449425.96	1402870.68	6.32E-14	3.12	13.20	272.186	1.53
Piperidine	34,329 86.0966 7.46 NA P	21866796.91	56688809.2	1.36E-13	2.59	12.87	86.0966	7.46
10-(beta-Dimethylaminopropionyl) phenothiazine	2354 297.1096 2.3 NA N	151118.01	308877.67	1.39E-12	2.04	11.86	297.11	2.3
Melatonin	4177 215.118 1.45 NA P	1536816.2	4636613.02	5.92E-11	3.02	10.23	215.118	1.45
Dibutyl sulfosuccinate	2170 309.1 4.1 NA N	100243.05	400677.00	9.32E-11	4.00	10.03	309.1	4.1
N-heptanoyl-homoserine lactone	3069 214.1436 1.77 NA P	130167.86	272694.11	4.54E-09	2.09	8.34	214.144	1.77
2-Acetylpyrrolidine	3642 114.0917 1.53 NA P	5846166.59	12053728.14	8.39E-09	2.06	8.08	114.092	1.53
Dibutyl sulfosuccinate	8892 309.1 4.22 NA N	114923.23	382686.68	5.01E-08	3.33	7.30	309.1	4.22
Gluconolactone	1898 177.0388 1.83 NA N	2818207.46	6947468.86	2.29E-07	2.47	6.64	177.039	1.83
3-Indoleacetonitrile	4957 157.0761 1.45 NA P	324709.14	744430.84	1.39E-06	2.29	5.86	157.076	1.45
Pentanamide	41,401 102.0912 1.97 NA P	53199585.96	153718573.2	1.96E-06	2.89	5.71	102.091	1.97
PYRROLIDINE-2-CARBALDEHYDE	10,219 100.0756 1.99 NA P	784390.43	2159792.49	2.77E-06	2.75	5.56	100.076	1.99
Erucin	9482 160.0268 7.09 NA N	212884.86	433015.68	2.77E-06	2.03	5.56	160.027	7.09
2-Dehydro-D-xylonate	9374 163.0232 1.79 NA N	3557348.17	9730937.79	5.43E-06	2.74	5.27	163.023	1.79
L,L-Cyclo(leucylprolyl)	2796 211.1441 1.54 NA P	964539.33	2463282.94	1.45E-05	2.55	4.84	211.144	1.54
Acetoxy-6-gingerol	11,599 337.2015 1.35 NA P	91213.95	204863.25	3.71E-05	2.25	4.43	337.202	1.35
4-Pyridoxolactone	17,622 164.0359 2.76 NA N	1603488.18	4054200.12	5.04E-05	2.53	4.30	164.036	2.76
3-Deaza-2’-deoxyadenosine	15,641 249.0972 1.89 NA N	118347.38	244849.99	0.00016379	2.07	3.79	249.097	1.89
1-hydroxyquinoline	26,405 146.0604 1.9 NA P	153524.47	439734.84	0.000497011	2.86	3.30	146.06	1.9
2,5-dihydro-2,4-dimethyloxzaxole	40,029 100.0758 3.4 NA P	245702.28	2407164.498	0.001091549	9.80	2.96	100.076	3.4
(R)-3-Hydroxy-5-phenylpentanoic acid	649 193.0886 1.72 NA N	2706828.94	5587170.165	0.001804092	2.06	2.74	193.089	1.72
Asparaginyl-Aspartate	15,831 246.0735 3.53 NA N	523376.61	1144979.991	0.007315639	2.19	2.14	246.074	3.53
4-Pyridoxic acid	8339 164.0333 1.76 NA N	270610.22	866546.7403	0.020716043	3.20	1.68	164.033	1.76
2-Hydroxy-3-morpholinopropanesulfonic Acid	10,291 224.0598 3.48 NA N	228396.41	649190.9916	0.036567075	2.84	1.44	224.06	3.48
N-Dimethyl-2-aminoethylphosphonate	1850 154.0633 3.13 NA P	246486.34	593936.6879	0.07293061	2.41	1.14	154.063	3.13

**Table 3:** Metabolites concentrations decreased from T1 to T4

Metabolite Name	Mean Baseline	Mean Time 4	Baseline vs. Time 4 *p* value	Time4/Baseline (Fold Change)	Log FC	Log p Value	Metabolite	M/Z	RT	Polarity
9-OxoODE	807316.8484	213604.3571	4.54E-09	0.264585531	-0.57743391	8.342944147	816	293.21	1.39	N
methyl 9,12-dihydroxy-13-oxo-10- octadecenoate	240295.5445	106017.2477	7.37E-08	0.441195229	-0.35536919	7.132532512	894	341.23	1.36	N
(±)4-HDoHE	564089.4378	60787.68784	0.000000476	0.1077625	-0.96753234	6.322393047	811	343.23	1.38	N
3-Hydroxy-10’-apo-b,y-carotenal	8757737.728	4270213.094	0.00000388	0.487593169	-0.31194239	5.411168274	638	391.26	1.36	N
3-HYDROXYPHENYLACETATE	6233095.228	1064125.687	0.00000388	0.170721872	-0.76771084	5.411168274	719	151.04	1.46	P
Sorbitan palmitate	3174643.67	1483053.062	0.0000504	0.467155755	-0.3305383	4.297569464	939	401.29	1.35	N
all-trans-8’-Apo-beta-carotenal	2287016.498	1067706.912	0.000122764	0.466855798	-0.33081724	3.910928969	1051	415.3	1.35	N
Tetracosapentaynoic acid	439102.5801	185276.7118	0.000217585	0.421944029	-0.37474515	3.662371048	1000	347.2	1.41	N
20-carboxy Arachidonic Acid	152672.5204	71023.83458	0.00037903	0.465203786	-0.33235676	3.421326415	9882	335.22	1.38	P
2-Pyrrolidinone, 4-(2-morpholinoethyl)-3,3-diphenyl-	140739.3866	61074.46065	0.005859125	0.43395429	-0.36255601	2.232167237	4133	349.2	1.42	N
5-ethyl-2-nitro-9 h-carbazole	1231036.119	323524.5459	p > 0.05	0.262806705	-0.58036356	0.810493755	6643	239.08	1.84	N
Phenylalanine	247381.9061	119854.5344	p > 0.05	0.484491919	-0.31471346	0.080963844	15,253	166.09	1.47	P

## Discussion

To our knowledge, this is one of the first studies to examine serum metabolite changes in women with early-stage BC from pre-chemotherapy to one year into survivorship. Our findings are a first step to a broader understanding of the biological changes associated with breast cancer treatments and survivorship. The metabolic changes in women between prior to chemotherapy and in survivorship were associated with lysine degradation, branched-chain amino acid synthesis, linoleic acid metabolism, tyrosine metabolism and biosynthesis of unsaturated fatty acids as the top 5 metabolic pathways. The biosynthesis of unsaturated fatty acids and linoleic acid were downregulated at T4 while branched-chain amino acid synthesis and lysine degradation were upregulated and tyrosine metabolism was differentially regulated. Levels of linoleic acid (LA), an omega-6 fatty acid and one of the essential polyunsaturated fatty acids (PUFAs) that are needed for cellular growth, were downregulated at T4. A pooled analysis of 20 studies in 39,740 subjects from 10 countries showed that higher plasma levels of LA are associated with a 43% reduced risk of diabetes, confirming other observations indicating an inverse relationship between dietary LA and the risk for T2D, which is, on the opposite, positively correlated with dietary saturated fatty acid [13]. Linoleic acid is first converted to gamma linolenic acid before ultimate conversion to arachidonic acid (20:4 n-6). Our data showed a reduction in both linolenic acid and arachidonic acid as well as metabolites associated with the arachidonic acid cascade (13-HODE, 20-carboxy arachidonic acid, and 2-hydroxy-9Z,12Z,15Z-octadecatrienoic acid). In addition to lower linoleic acid metabolites, we also observed a decrease in docosahexaenoic acid (22:6 n-3) an omega-3 PUFA. Thus, in general we observed a downregulation in fatty acid metabolism in survivorship. Elevated/increased dietary intake or tissue levels of LA is associated with a reduced incidence of cardiovascular diseases (mainly coronary artery diseases) and of new onset metabolic syndrome or type 2 diabetes. In addition, increased levels of steroid hormone synthesis metabolite lysophosphatidic acid have been implicated in the activation of signaling pathways regulating inflammation, oxidative stress and cell proliferation [18].

Branched-chain amino acid (BCAA) synthesis was upregulated: increased BCAA concentrations are found in various insulin-deficient and -resistant states, especially diabetes and obesity. An analysis of 139 serum metabolites found higher branched-chain AAs (BCAAs) leucine, isoleucine and valine, aromatic AAs 140 (AAAs) phenylalanine and tyrosine, as well as alanine, methionine, glutamate, lysine and proline in 141 volunteers with type 2 diabetes mellitus relative to non-diabetic controls. A recent study testing supplementation of both omega-3 and omega-6 PUFAs in breast cancer survivors found that omega-6 supplementation significantly reduced cancer related fatigue (CRF) [14]. Both supplements resulted in a decrease in the majority of serum AAs, including BCAAs and AAAs indicating the modifiability with nutritional intake [15]. Greater 10-year increases of branched-chain amino acids (BCAAs), diglyceride-/triglyceride-fragments, phosphatidylethanolamines, some vitamins, and bile acids were associated with higher type 2 diabetes risk and BCAAs have been linked to type 2 diabetes [16]. In cancer, BCAAs are involved in stimulating protein growth in tumors through activation of mTORC1 [17]. Abnormal regulation of FAs has been linked with cardiovascular conditions such as myocardial infarction and hypertrophy [18]. In addition, we noted decreased arachidonic acid metabolism at the one-year mark. An alteration in arachidonic acid (AA) metabolism is seen in the form of increased formation of pro-inflammatory eicosanoids and decreased production of anti-inflammatory lipoxins, type 2 diabetes mellitus, hypertension and endothelial dysfunction that are common with increasing age and aging-associated conditions. In all these conditions, the elevated levels of BCAAs and arachidonic acid metabolism noted in this study in are in the direction of heightened inflammatory responses.

Lysine degradation was upregulated: lysine is inversely correlated with numerous markers of inflammation including endotoxin, TLR-4, and IL-6. Moreover, acetylation of lysine is seen in states of insulin resistance and is also thought to play a role in immunomodulation. This inverse correlation may indicate an attempt to blunt the inflammatory response, leading to a depletion in lysine. Lysine and 2-AAA have also been implicated in the development of other CVD risk factors, such as obesity and metabolic syndrome. Previous studies have shown that circulating 2-aminoadipic acid (2-AAA) levels were associated with obesity and metabolic syndrome and had the ability to predict the risk of future T2D [19]. Aminoadipate is generated by lysine degradation and may also serve as a substrate for enzymes downstream of tryptophan metabolism. The current and previous findings collectively suggest that the mechanism behind metabolic syndrome and insulin resistance involves alterations in these metabolic pathways, distinct from pathways of BCAAs [20]. Lysine degradation attributed to microbiome changes in liver metabolism or the microbiome. In an animal model, lysine supplementation was shown to increase the spread of ER + breast cancer cells. Pipecolic acid (a metabolite of lysine) has been implicated in other cancers such as prostate and renal cell carcinoma, but its connection to breast cancer survivorship is currently unknown [21, 22]. In addition to perturbations in lysine regulation, we noted downregulated biosynthesis of unsaturated fatty acids: One study in women undergoing treatment for breast cancer with serum samples collected prior to chemotherapy (baseline; n = 50), just after the fourth cycle of chemotherapy (chemo-4; n = 40), and 6 months after beginning chemotherapy (6 M; n = 34) suggested a dysregulation of PUFAs post-chemotherapy with higher serum PUFAs associated with lower inflammation before, during, and after chemotherapy [23] suggests that n-3 polyunsaturated fatty acid (n-3 PUFA) supplementation during cancer chemotherapy may improve outcomes related to chemotherapy tolerability, [24].

## Limitations

There are some limitations that should be noted in this study. First, there was no control sample of women who were not undergoing treatment for early-stage breast cancer and our sample was a convenience sample from one state in the United States. However, the longitudinal design of the current study was sufficient for noting intra-person and group level differences and we had significant diversity in the sample. Secondly, variables related to treatment regimen were not standardized, given the multiple different treatment regimens for early-stage breast cancer. The metabolite data were highly non-normal even after applying transformations and therefore common assumptions for most statistical models such as normality and equal variance are not satisfied. Therefore, we chose to use a rank-based approach, namely, Friedman’s ANOVA, which does not require such assumptions. For that reason, we did not adjust for demographic or lifestyle variables. However, the naturalistic design depicts the effects of the usual treatment regimens in an academic medical center. And, while metabolomics can be affected by fasting state, our participants, given their other health-care challenges during cancer treatment, were not advised to fast prior to blood draws, which were coordinated with usual care health appointments to decrease participant burden.

## Conclusion

In this study, we have shown that the circulating metabolomic profiles significantly changed in women with breast cancer from prior to chemotherapy to one-year survivorship. We also show that many of these changes relate to molecules involved in heightened cardiometabolic risk. We conclude that LC–MS based non-targeted technology could be useful in further understanding risk factors for excessive morbidity and mortality in breast cancer survivors related to cardiovascular and metabolic risk factors. Significant differences in various metabolite levels were found over time. Perturbations in the metabolic profiles of women receiving chemotherapy for early stage breast cancer may not only serve as objective biomarkers for prediction and interventions to improve health outcomes associated with early stage BC and chemotherapy, but may also influence survivorship. Further elucidating them could have implications for innovative individualized treatment options to alleviate symptoms associated with treatment and survivorship. Many of the metabolite changes may be potentially modifiable with diet and nutritional supplementation. Our study highlights the need of larger-scale longitudinal metabolomic studies to provide more detailed understanding of metabolism of common, adverse outcomes in breast cancer survivors. These metabolic changes may have an effect on long-term morbidity after treatment, which warrants further investigation. These data merit the pursuit of further longitudinal study of the metabolite changes associated with breast cancer treatments that may be contributing to accelerated morbidity associated with adverse metabolic and cardiovascular outcomes in breast cancer survivors.

## Data Availability

The data analyzed during the study is available from the corresponding author upon reasonable request.
